# Dietary knowledge assessment among the patients with type 2 diabetes in Madinah: A cross-sectional study 

**DOI:** 10.12688/f1000research.131518.2

**Published:** 2024-01-08

**Authors:** Mashael Alharbi, Mansour Alharbi, Amal Surrati, Mashael Alhilabi, Ayed alrashdi, Majedah Almokhalafi

**Affiliations:** 1 Ministry of Health, Riyadh, Saudi Arabia; 2 Ministry of Health, , Al-Madinah Al-Munawarah, Saudi Arabia; 3 Family and Community Medicine Department, College of Medicine, Taibah University, Madinah, Saudi Arabia; 4 Ministry of Health, King Abdullah Medical City,, Riyadh, Saudi Arabia; 5 Riyadh Health Affairs, Ministry of Health, Riyadh, Saudi Arabia; 6 Madinah Health Cluster, First Network, Riyadh Health Affairs, Ministry of Health, Riyadh, Saudi Arabia

**Keywords:** Type 2 diabetes mellitus, dietary knowledge, awareness, Saudi Arabia

## Abstract

**Background:**

There is a huge burden of nutrition-related non-communicable diseases, and diabetes is one of the leading chronic nutrition-related diseases affecting more than 500 million people globally. Collecting information regarding the awareness of dietary and nutrition knowledge among diabetic patients is the first step to developing a disease prevention program. Thus, this study primarily aims at assessing the dietary awareness of diabetes patients attending the diabetic centre in Madinah governorate, Saudi Arabia.

**Methods:**

The study was started in November 2020 and ended in October 2021. The study participants (315) were type 2 diabetes mellitus (T2DM) patients attending a diabetic centre in Madinah, Saudi Arabia. A self-prepared dietary knowledge questionnaire (DKQ) was used in this research. The variables include balanced diet, food type, food choice, carbohydrate, protein, and fat. Knowledge score was, and the total score was levelled/categorized into ‘good’, ‘average’, and ‘poor’. Data were analysed by SPSS v.26.

**Results:**

The study results identified the current knowledge of T2DM patients about different dietary items. The knowledge score of 62.2% of participants showed an average level of dietary knowledge, which is statistically significant. When we separately evaluated their understanding of different dietary components, we found that T2DM patients had poor knowledge of carbohydrates (30.15%), fat, food choices (47.7%), and type (34.6%). However, they had acceptable knowledge of proteins (56.5%).

**Conclusion:**

Our participants exhibited acceptable knowledge about proteins but poorer knowledge of other food groups. A healthy, well-balanced diet is essential for excellent glycaemic control. Educating and arranging a health education program regarding dietary knowledge is recommended, specially designed for diabetic patients so that patients can opt for a healthier lifestyle

## Introduction

Today’s world has a huge burden of nutrition-related non-communicable diseases. ^1^ Diabetes is one of the leading chronic nutrition-related diseases affecting more than 500 million people globally (around 6.3% of the world’s population). ^2^^,^^3^ Diabetes is the ninth primary cause of mortality. ^2^ It is also estimated that over 1.5 million deaths were directly caused by diabetes alone. ^4^ However, the main concern raised recently is that greater than one-third of diabetes-related mortalities occur in individuals younger than 60. ^5^ The International Diabetes Federation (IDF) for the Middle East and North Africa (MENA) recently estimated that diabetes prevailed at 18% in Saudi Arabia alone. ^3^

Studies have shown a continuous upward trend in the prevalence of diabetes. ^2^ There is also an increased burden of human suffering as measured by disability-adjusted life years (DALYs) because of diabetes. However, many countries have implemented a significantly high budget for health care expenditure. ^6^ Non-modifiable risk factors like age and familial history can be one of the causes of the continuous upward trend. ^7^ At the same time, modifiable risk factors include increased body mass index due to processed high caloric unhealthy diet and physical inactivity for type 2 diabetes mellitus (T2DM). ^4^^,^^7^

Recent analysis has reported that for the management of diabetes, only pharmacologic approaches are not sufficient. ^2^ The management of diabetes is a multifactorial process involving demographic and social factors that influence the treatment outcomes. ^8^ There are also patient-related factors, such as self-care behaviours, that have a strong influence on glycaemic intolerance. ^9^ Blood glucose monitoring, diabetes-related knowledge, and dietary knowledge all come under self-care behaviours. ^10^ Similarly, Dietary monitoring is necessary to predict patients’ quality of life, nutritional status, and risk factors to avoid chronic diabetes-related complications. ^10^

Early onset T2DM usually occurs in <45 years; its main contributor include obesity, dyslipidaemia, smoking, and a sedentary lifestyle. ^11^ Simple lifestyle modifications are effective in preventing or delaying complications in T2DM patients. ^4^ Dietary modifications and nutrition knowledge are the cornerstones of diabetes type 2 management. ^12^ Eating habits are directly related to dietary knowledge and significantly contribute to glycaemic control. ^12^ Nutrition knowledge helps individuals with diabetes to optimize their quality of life. ^13^

Patients with T2DM often face difficulty identifying the recommended diet, including its quality and quantity. The collection of food and dietary habits is affected by the knowledge of a patient concerning a suggested diet. ^14^ A study on dietary knowledge among T2DM patients conducted in the Almajamah area of Saudi Arabia found poor awareness, attitudes, and practices toward healthy dietary habits. Only one-sixth of the studied population followed a strict diabetic diet that represented a poor diabetic control level. ^15^ Non-adherence to dietary plans with inadequate knowledge is facing in many countries, ^16^ which in turn is associated with inadequate family support ^17^ and decreased motivation. ^18^ Diabetes in < 60 years of age is most prevalent in low-and middle-income countries, while in high-income countries, this is mostly found in the ageing population (>60 years of age). ^15^ Collecting information regarding the awareness of dietary and nutrition knowledge among diabetic patients is the first step to developing a disease prevention program. Although there is an increased prevalence of T2DM, studies have shown that the dietary knowledge among patients is insufficient. ^15^ Knowing the level of knowledge and perception of dietary habits in T2DM patients helps provide them with more efficient and beneficial education. Therefore, this study aims to assess the dietary knowledge among diabetic patients attending the diabetic centre, Madinah governorate, Saudi Arabia.

## Methods

### Ethical statement

Ethical approval was obtained from Research and Studies Department of Health Affairs, Ministry of Health, Madinah, Saudi Arabia (IRB#544). All patients who gave voluntary written informed consent for participation before data collection were included in the study. The study participants’ information was kept confidential and anonymous.

### Study design

This study was a cross-sectional descriptive, single-centre study conducted at Al-Madinah Al Munawarah, the second holiest city in Islam, in the Hejaz region of western Saudi Arabia. The study was started in November 2020 and ended in October 2021.

### Study population

The study population consist of patients with Type 2 Diabetes mellitus attending a diabetic centre in Madinah, Saudi Arabia. The inclusion criteria were type 2 diabetic patient, > 18 years of age, and the participation was voluntary. Those who were type 1 diabetic patients, < 18 years old and those with mental impairment or who did not consent to participate were excluded from the study. A systematic random sampling technique was utilized for participant recruitment and data collection.

### Sample size

Population size: the total number of type 2 diabetic patients attending the diabetic centre.

Confidence limit: 5%

confidence interval: 95%

Diabetes prevalence in KSA: 18% ^3^

Using Epi info to calculate the sample size, the calculated sample size was 227. However, it was increased to 315 to overcome the possibility of outliers and missing data (if any).

### Study tools

The self-prepared Dietary Knowledge questionnaire (DKQ) used in this research consisted of multiple-choice questions (MCQs) prepared after an extensive literature review. The questionnaire was face validated by faculty members of the university. Back-to-back translations (Arabic and English) were made by the native Arabic speaker and reviewed by the corresponding author to improve the clarity of the questions. ^30^^,^^31^ The pilot study was also conducted, and the changes were done according to responses and unclear questions. The researcher interviewed ten patients (started 15 September 2020 and ended in 20 September 2020) from outside the selected class to test the feasibility of the questionnaire, the time taken to finish the interview. These ten cases were not included in main study. Those questions that were unclear or that did not cover the study concept were modified accordingly. The questionnaire was adapted from a published article, ^19^ which showed the Cronbach Alpha of that questionnaire was 0.869.

### Data collection technique

The questionnaire was distributed online through google survey forms. The completed version of the forms was saved in the secured email (which was only assessed by the study supervisor) and collected after the required sample size was achieved. Selection bias is common in population-based studies; to overcome this we sent invitations to all the relevant participants who attended the clinics. Therefore, who those who were willing to participate filled the online questionnaire.

The questionnaire was self-administered and consisted of 19 questions with multiple choices. Variables include knowledge about a balanced diet, food type, food choice, carbohydrates, proteins, and fats. However, only one response is correct. The correct response was coded 1, and all others were coded 0. Therefore, the total score = 19 (< 8 = poor level, 8–12 = average level, and > 12 good knowledge level). The higher score of the individual corresponded to higher dietary knowledge. However, each variable’s quantitative scores were categorized into 3: <50% as poor knowledge, 50-75% as acceptable, and >75% as adequate knowledge. ^12^^,^^20^

### Data entry and analysis

All data were analysed by SPSS software version 26. The normality of the quantitative variables was checked. The frequency (percentage) of correct responses for each variable was presented. Outliers were detected through Z-scores. Knowledge score was, and the total score was levelled/categorized into ‘good’, ‘average’, and ‘poor’. One-sample T-test was performed to evaluate the dietary knowledge of T2DM individuals. A p-value of <0.05 is considered statistically significant.

## Results

The overall frequency and percentage of correct responses were presented against each item in Table 1. ^29^ The dietary knowledge questions were also categorized into food choices, knowledge about carbohydrates, fats/oil, protein, and type of food. Most patients select correct food choices for diabetes (85.4%). While very few (19%) know which of the food they can freely eat. Almost half of the participants knew how to treat low blood sugar (42.5%). The median knowledge score of participants for the food choices was 1(0-3), corresponding to poor knowledge (47.3%) about food choices for T2DM.

**Table 1.** Dietary knowledge of type 2 diabetes mellitus (T2DM) patients (n = 315). ^29^

S.#	Questions	Correct response n (%)
1	A balanced diet contains?	236 (74.9)
2	The diet of diabetics is?	269 (85.4)
3	Which of the following is highest in carbohydrates?	123 (39)
4	Which of the following is highest in fat?	162 (51.4)
5	Which of these foods contains the highest percentage of proteins?	224 (71.1)
6	Which of these foods contains the highest percentage of sugar?	54 (17.1)
7	Which of these foods contains the highest percentage of fat?	232 (73.7)
8	Which of these for the following foods contains high cholesterol?	59 (18.7)
9	Bread, cereals, rice, and pasta rich?	206 (65.4)
10	Which of the following food is a complete source of protein?	238 (75.6)
11	Whole grain foods such as brown rice and whole wheat bread are better options than white rice and white bread because whole grains contain:	192 (61.0)
12	Eating too many sugary foods leads to?	249 (79)
13	Which of these foods can you freely eat?	60 (19)
14	What is the effect of unsweetened fruit juice on blood glucose?	145 (46)
15	What to use to treat low blood sugar:	134 (42.5)
16	HbA1c test has some relationship with your diet?	264 (83.8)
17	Food that contains fats and oils is a rich source of?	280 (88.9)
18	Which of the following has the highest glycaemic index?	237 (75.2)
19	Do you think that justified consumption of vitamins, minerals, carbohydrates, fat, and protein has a direct effect on outcomes of diabetes mellitus?	205 (65.1)

Regarding the knowledge about dietary group carbohydrates, two-fifths of the participants correctly identified the food with the highest carbohydrate percentage. Around two-thirds of the participants knew that bread, cereals, rice, and pasta are a rich source of carbohydrates. Many participants did not know which food contained the highest amount of sugar (82.9%). However, most participants (61%) correctly responded about whole grains foods. The majority know that eating too much sugar will lead to diabetes (80%). The median knowledge score about carbohydrates was three (0-5), while the percentage showed poor overall knowledge of carbohydrates (30.15%).

Regarding dietary knowledge about protein, most participants correctly identified the food with the highest protein percentage (71.1 %), and most knew the complete source of protein (75.6%). The median knowledge score of protein was 2 (0-2), and the percentage showed an almost acceptable level of knowledge of protein (56.5%). Concerning the fat and oil group, most of the patients know food with the highest percentage of fat (73.7%) and which is highest in fat (51.4%). Only one fifth of the participants knew the food product with a high cholesterol level. While a majority of the patients correctly identified that fat is a rich source of calories (88.9%). The median knowledge score of fat was 2 (0-3), whereas, in terms of percentage, T2DM patients have poor knowledge of fat (47.7%). There were various statements on a selection of food types. The median knowledge score of food type was four (0-5), with the percentage showing that T2DM patients have poor knowledge of food type (34.6%).

**Figure 1.**
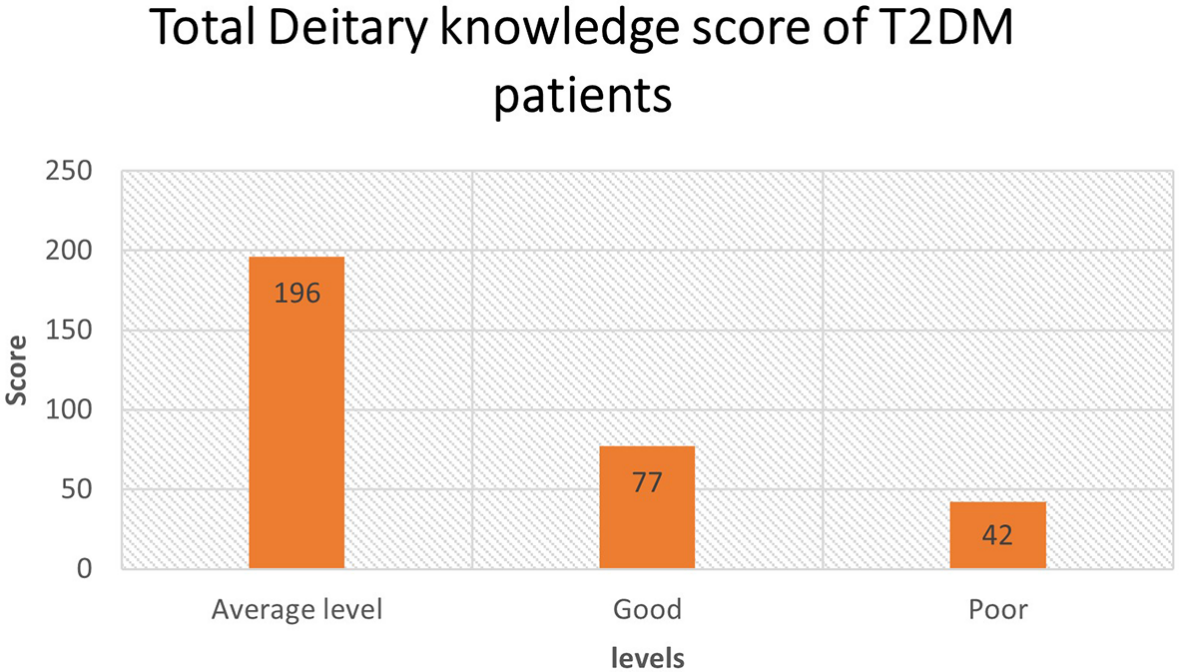
Total dietary Knowledge score of type 2 diabetes mellitus (T2DM) patients.

The study results showed that all the z-scores of dietary knowledge score were less than three, whereas the minimum z-score was -2.85 and the maximum was 2.08. There was no outlier detected in the dietary knowledge variable. The mean dietary knowledge score (total 19 items) was 10 (8-12), with a minimum of three and a maximum of 16. However, most of the study participants (62.2%) showed average dietary knowledge ( Figure 1). One sample T-test statistics confirmed that the T2DM patient’s average level of dietary knowledge is statistically significant (p < 0.0006).

## Discussion

The study results identified the current knowledge of T2DM patients about different dietary items. The knowledge score of 62.2% of participants showed an average level of dietary knowledge, which is statistically significant. When we separately evaluated their awareness of different dietary components, we found that T2DM patients have poor knowledge about carbohydrates, fats, food choices, and food types. However, they had acceptable knowledge of proteins.

Different studies around the world showed various levels of dietary knowledge among type 2 diabetic patients. In agreement with our study, another study conducted on T2DM patients in Iran showed average levels of dietary knowledge. ^21^ In contrast, a study reported inadequate dietary knowledge among T2DM females of Amman, Jordan. ^20^ A study conducted in Ethiopia found a poor level of dietary knowledge and practices. ^22^

Our study results showed that the majority of the participants answered that they know what a diabetic diet (85.4%) is; however, they have poor knowledge regarding individual food components and what should be used with hypoglycaemia (42.5%). Comparing our results with another study, only 43.9% of individuals know the recommended meal for diabetes/day, and only 44.5% know what should be used with hypoglycaemia. ^20^

Carbohydrates are an important macronutrient that affects the postprandial glycaemic index. Another study conducted among Emirati and Omani adults concluded that individuals have insufficient knowledge of carbohydrates. ^23^ Likewise, another Saudi study showed participants had poor knowledge about carbohydrates but had good dietary knowledge regarding other good groups, including fat and protein. ^14^ Most participants correctly identified the main carbohydrate source in the diabetic diet, but most did not know the food with the highest sugar content. Global estimates from 165 countries found a strong positive association between diabetes prevalence and per capita sugar consumption. ^24^

During the last decades, many countries have experienced a major shift in food choices and nutritional transition from the Mediterranean diet to more processed/junk foods. Likewise, lack of physical activity, urbanized lifestyle, and increased food consumption cause an increase in nutritional problems with a high prevalence of obesity that triggers many chronic diseases. ^20^ In Saudi Arabia, food choices and portion sizes with a sedentary lifestyle were dramatically increased, resulting in a high prevalence of obesity and diabetes. Moreover, they also consume too many sugary drinks. ^25^ A recent randomized controlled trial of diabetic patients demonstrated that simplified diabetes nutrition education greatly impacts glycaemic control. ^26^

### Strengths and limitations

The first limitation is the study's cross-sectional nature, which limits the temporal association between disease and exposure. Secondly, the study participants were only recruited from a single centre; therefore, the generalizability of the study results should be interpreted with caution. Thirdly, the sociodemographic data were not collected for this study hence, the comparison and difference of knowledge across different socioeconomic groups was not evaluated. However, there are also some strengths of the study. First, a valid questionnaire was used, and study participants were recruited through systematic random sampling. Second, the sample size was calculated, and a maximum number of participants was recruited to overcome the missing data.

## Conclusion

A healthy, well-balanced diet is essential for excellent glycaemic control. ^27^ Appropriate self-care and effectual metabolic regulation control prevent hypoglycaemia, other metabolic complications, and ketoacidosis. ^27^ Nutrition plays a key role; food with high fibre, low fat, and limited carbohydrate should be taken at regular intervals. ^25^ All food groups should be included in the daily diet. ^28^ Our participants showed acceptable protein knowledge but poorer knowledge of other food groups. It is not an easy job to increase the awareness and knowledge of nutrition and a healthy diet in diabetic individuals in our society. Self-dietary management is the first and foremost step in providing knowledge and skills to people with diabetes concerning treatment, medications, and nutritional aspects as defined by American Diabetes Association (ADA). ^25^ A healthy diet is considered an integral part of diabetes management. Likewise, a healthy lifestyle, regular exercise, weight management, and a well-balanced diet can improve diabetic patients' health. Educating and arranging a health education program regarding dietary knowledge is recommended, specially designed for diabetic patients so that patients can opt for a healthier lifestyle. There is also a need to emphasize preventive measures with research based on an effective region-specific diet and nutritional knowledge that can be provided to everyone, including prediabetics and those with diagnosed diabetes.

## Data Availability

Figshare: Dietary knowledge assessment among the patients with type 2 diabetes in Madinah: A cross-sectional study. https://doi.org/10.6084/m9.figshare.22122656.v1. ^29^ The project contains the following underlying data:
• Data.xlsx. (Anonymised answers to questionnaire, correct answers – 1, incorrect answers - 0). Data are available under the terms of the Creative Commons Attribution 4.0 International license (CC-BY 4.0). Figshare: Dietary knowledge assessment among the patients with type 2 diabetes in Madinah: A cross-sectional study. https://doi.org/10.6084/m9.figshare.22123001.v1. ^30^ This project contains the following extended data:
• Nutritional Knowledge Questionnaire in Diabetics (English).docx. (Blank English version of the questionnaire) Data are available under the terms of the Creative Commons Attribution 4.0 International license (CC-BY 4.0). Figshare: Nutritional Knowledge Questionnaire in Diabetics (Arabic).docx https://doi.org/10.6084/m9.figshare.22123004.v1. ^31^ This project contains the following extended data:
• Nutritional Knowledge Questionnaire in Diabetics (Arabic).docx. (Blank Arabic version of the questionnaire) Data are available under the terms of the Creative Commons Attribution 4.0 International license (CC-BY 4.0). Figshare: STROBE checklist and flowchart for “Dietary knowledge assessment among the patients with type 2 diabetes in Madinah: A cross-sectional study”. https://doi.org/10.6084/m9.figshare.22083365.v1. ^32^ Data are available under the terms of the Creative Commons Attribution 4.0 International license (CC-BY 4.0).
