# Supplementary material for: Dietary knowledge assessment among the patients with type 2 diabetes in Madinah: A cross-sectional study 
Source: F1000Res. 2024 Jan 8;12:416. Originally published 2023 Apr 18. [Version 2] doi: 10.12688/f1000research.131518.2 (PMC10792270; doi:10.12688/f1000research.131518.2)
Supplement: Peer review history

# Reviewer report: Reviewer response for version 2

**DOI:** 10.5256/f1000research.160812.r236177

**Published:** 2024-01-16

**Authors:** Munazza Jawed

**Affiliations:** 1 School of Public Health, Dow University of Health Sciences, Karachi, Pakistan

good efforts. No further comments.

Is the work clearly and accurately presented and does it cite the current literature?

Yes

If applicable, is the statistical analysis and its interpretation appropriate?

Yes

Are all the source data underlying the results available to ensure full reproducibility?

Yes

Is the study design appropriate and is the work technically sound?

Partly

Are the conclusions drawn adequately supported by the results?

Yes

Are sufficient details of methods and analysis provided to allow replication by others?

Yes

Reviewer Expertise:

Epidemiology, Diabetes, Public health.

I confirm that I have read this submission and believe that I have an appropriate level of expertise to confirm that it is of an acceptable scientific standard.

# Reviewer report: Reviewer response for version 1

**DOI:** 10.5256/f1000research.144366.r178211

**Published:** 2023-12-19

**Authors:** Ashraf El-Metwally

**Affiliations:** 1 College of Public Health and Health Informatics, King Saud Bin Abdulaziz University for Health Sciences, Riyadh, Saudi Arabia

The document exhibited a high level of writing proficiency and holds significant relevance in the field of public health in the Kingdom of Saudi Arabia. The information presented within was clearly articulated and supported by accurate references to current scholarly works. The researcher employed suitable methodology throughout the study; however, it is noteworthy that the authors acknowledged a limitation that they didn’t collect sociodemographic data, which constitutes a substantial concern. It is strongly advised that future research endeavors incorporate sociodemographic questions, as these factors have the potential to influence statistical analysis and subsequent findings.

Is the work clearly and accurately presented and does it cite the current literature?

Yes

If applicable, is the statistical analysis and its interpretation appropriate?

Partly

Are all the source data underlying the results available to ensure full reproducibility?

Yes

Is the study design appropriate and is the work technically sound?

Yes

Are the conclusions drawn adequately supported by the results?

Yes

Are sufficient details of methods and analysis provided to allow replication by others?

Yes

Reviewer Expertise:

Public health, statistics, epidemiology.

I confirm that I have read this submission and believe that I have an appropriate level of expertise to confirm that it is of an acceptable scientific standard.

# Response

**Published:** 2023-12-30

**Authors:** Mashael Alharbi

**Affiliations:** Ministry of health, Riyadh, 12478, Saudi Arabia, Saudi Arabia

Dear Reviewer,

 Thank you for your kind review and acknowledgment of the article. We have made all the suggested changes as per your comments. Your comments really improved our article. Your feedback regarding incorporating the sociodemographic questions in future research are duly noted. While we aimed to provide a comprehensive overview, we understand the potential benefits of understand impact of different socioeconomic levels and these factors might have the potential to influence statistical analysis and subsequent findings.

# Reviewer report: Reviewer response for version 1

**DOI:** 10.5256/f1000research.144366.r195807

**Published:** 2023-08-30

**Authors:** Munazza Jawed

**Affiliations:** 1 School of Public Health, Dow University of Health Sciences, Karachi, Pakistan

I carefully read your manuscript titled “Dietary knowledge assessment among the patients with Type 2 diabetes in Madinah: A cross-sectional study”. The paper is generally well-written and structured and demonstrates smooth flow. The paper shows an interesting fact and will be a valuable addition relevant to its field and improve policymaking. The article does a good job of assessing the dietary knowledge among patients with Type 2 diabetes. The authors reported mostly patients with T2DM have inadequate knowledge of nutrition and food groups that are essential for excellent glycaemic control. However, I think the paper has some shortcomings regarding comparing its results. It would be best if the author compared it across different socio-demographic statuses (not collected), a major study limitation. The author used recent references.

 Minor comments:

 Abstract:

 Method section: change spelling of leveled to levelled.

 Conclusion last sentence, “it is recommended….” Change it to this “Educating and arranging a health education program regarding dietary knowledge is recommended, specially designed for diabetic patients so that patients can opt for a healthier lifestyle”.

 Introduction:

 1st paragraph, 4th line: delete word “alone”.

 2nd paragraph, 3rd line: Replace “Health care” to “healthcare”.

 3rd paragraph, 2nd line: remove “the” before treatment.

 4th paragraph, 1st line: change it to this “Early onset T2DM usually occurs in <45 years; its main contributor include obesity, dyslipidaemia, smoking, and a sedentary lifestyle”  

 4th paragraph, 2nd line: delete “shown to be”

 5th paragraph, 6th line: change “poor family support” to inadequate family support”

 5th paragraph, 8th line: replace aging to “ageing”

 Methods:

 Study design first sentence: replace it with this “This study was a cross-sectional descriptive study conducted at Al-Madinah Al Munawarah, the second holiest city in Islam, in the Hejaz region of western Saudi Arabia.”

 Study population: start sentence with “The study population”. 2nd line remove coma after patient. Change 3rd sentence to “Those who were type 1 diabetic patients < 18 years old and those with mental impairment or who did not consent to participate were excluded from the study”.  

 Study tools: 3rd line, replace “done” to “made”.

 5th line, the numeral 10 is used instead of the word spelled out. Consider spelling out the number.

 6th line, replace it with this sentence “selected class to test the feasibility of the questionnaire, the time taken to finish the interview”.

 8th line, remove first word “that”.

 10th line, google to Google.

 11th line, change sentence to “Selection bias is common in population-based studies; to overcome this we sent invitations to all the relevant participants who attended the clinics.

 12th line, change sentence to “Therefore, who are only willing to participate filled out the online questionnaire”.

 Data entry:

 2nd line, response to responses

 Results:

 Two third to two-third

 One fifth to one-fifth

 Last paragraph, 3rd line, change these sentences “with a minimum of three and a maximum of 16”. “However, most of the study participants (62.2%) showed average dietary knowledge”.

 Discussion:

 1st paragraph, 4th line, fat to fats, type to types.

 4th paragraph, 3rd line, change sentence “likewise, another Saudi study…” to “Likewise, another Saudi study showed participants had poor knowledge”.

 Conclusion:

 3rd line: change sentence to “All food groups should be included in the daily diet”

 9th line: change sentence “It is recommended to…” to “Educating and arranging a health education program regarding dietary knowledge is recommended, specially designed for diabetic patients so that patients can opt for a healthier lifestyle”.

 Overall: For consistency, consider replacing words with the British English spelling. For example, leveled to levelled, center to centre, behavior to behaviour, fiber to fibre.

Is the work clearly and accurately presented and does it cite the current literature?

Yes

If applicable, is the statistical analysis and its interpretation appropriate?

Yes

Are all the source data underlying the results available to ensure full reproducibility?

Yes

Is the study design appropriate and is the work technically sound?

Partly

Are the conclusions drawn adequately supported by the results?

Yes

Are sufficient details of methods and analysis provided to allow replication by others?

Yes

Reviewer Expertise:

Epidemiology, Diabetes, Public health.

I confirm that I have read this submission and believe that I have an appropriate level of expertise to confirm that it is of an acceptable scientific standard, however I have significant reservations, as outlined above.

# Response

**Published:** 2023-12-30

**Authors:** Mashael Alharbi

**Affiliations:** Ministry of health, Riyadh, 12478, Saudi Arabia, Saudi Arabia

Abstract:

 Method section: change spelling of leveled to levelled.

 Conclusion last sentence, “it is recommended….” Change it to this “Educating and arranging a health education program regarding dietary knowledge is recommended, specially designed for diabetic patients so that patients can opt for a healthier lifestyle”.

 Introduction:

 1st paragraph, 4 ^th^ line: delete word “alone”.

 2 ^nd^ paragraph, 3 ^rd^ line: Replace “Health care” to “healthcare”.

 3 ^rd^ paragraph, 2 ^nd^ line: remove “the” before treatment.

 4 ^th^ paragraph, 1 ^st^ line: change it to this “Early onset T2DM usually occurs in <45 years; its main contributor include obesity, dyslipidaemia, smoking, and a sedentary lifestyle” 

 4 ^th^ paragraph, 2 ^nd^ line: delete “shown to be”

 5 ^th^ paragraph, 6 ^th^ line: change “poor family support” to inadequate family support”

 5 ^th^ paragraph, 8 ^th^ line: replace aging to “ageing”

**Response: All the above suggested changes have been done in the manuscript.**

 Methods:

 Study design first sentence: replace it with this “This study was a cross-sectional descriptive study conducted at Al-Madinah Al Munawarah, the second holiest city in Islam, in the Hejaz region of western Saudi Arabia.”

 Study population: start sentence with “The study population”. 2 ^nd^ line remove coma after patient. Change 3 ^rd^ sentence to “Those who were type 1 diabetic patients < 18 years old and those with mental impairment or who did not consent to participate were excluded from the study”.  

 Study tools: 3 ^rd^ line, replace “done” to “made”.

 5 ^th^ line, the numeral 10 is used instead of the word spelled out. Consider spelling out the number.

 6 ^th^ line, replace it with this sentence “selected class to test the feasibility of the questionnaire, the time taken to finish the interview”.

 8 ^th^ line, remove first word “that”.

  10 ^th^ line, google to Google.

 11 ^th^ line, change sentence to “Selection bias is common in population-based studies; to overcome this we sent invitations to all the relevant participants who attended the clinics.

 12 ^th^ line, change sentence to “Therefore, who are only willing to participate filled out the online questionnaire”.

 Data entry:

 2 ^nd^ line, response to responses

**Response: All the above suggested changes in method section have been made in the manuscript.**

 Results:

 Two third to two-third

 One fifth to one-fifth

 Last paragraph, 3 ^rd^ line, change these sentences “with a minimum of three and a maximum of 16”. “However, most of the study participants (62.2%) showed average dietary knowledge”.

**Response: All the above suggested changes have been made to the manuscript.**

 Discussion:

 1 ^st^ paragraph, 4 ^th^ line, fat to fats, type to types.

 4 ^th^ paragraph, 3 ^rd^ line, change sentence “likewise, another Saudi study…” to “Likewise, another Saudi study showed participants had poor knowledge”.

**Response: All the above suggested changes in discussion have been made to the manuscript.**

 Conclusion:

 3 ^rd^ line: change sentence to “All food groups should be included in the daily diet”.

 9 ^th^ line: change sentence “It is recommended to…” to “Educating and arranging a health education program regarding dietary knowledge is recommended, specially designed for diabetic patients so that patients can opt for a healthier lifestyle”.

**Response: All the above suggested changes in conclusion have been made to the manuscript.**

 Overall: For consistency, consider replacing words with the British English spelling. For example, leveled to levelled, center to centre, behavior to behaviour, fiber to fibre.

**Response: All the above suggested changes have been made throughout the manuscript.**
